# The time course of salience: not entirely caused by salience

**DOI:** 10.1007/s00426-020-01470-6

**Published:** 2021-02-18

**Authors:** Alexander Krüger, Ingrid Scharlau

**Affiliations:** Faculty of Arts and Humanities, Paderborn University, Warburger Straße 100, 33098 Paderborn, Germany

**Keywords:** Attention, Visual perception

## Abstract

Visual salience is a key component of attentional selection, the process that guards the scarce resources needed for conscious recognition and perception. In previous works, we proposed a measure of visual salience based on a formal theory of visual selection. However, the strength of visual salience depends on the time course as well as local physical contrasts. Evidence from multiple experimental designs in the literature suggests that the strength of salience rises initially and declines after approximately 150 ms. The present article amends the theory-based salience measure beyond local physical contrasts to the time course of salience. It does so through a first experiment which reveals that—contrary to expectations—salience is not reduced during the first 150 ms after onset. Instead, the overall visual processing capacity is severely reduced, which corresponds to a reduced processing speed of all stimuli in the visual field. A second experiment confirms this conclusion by replicating the result. We argue that the slower stimulus processing may have been overlooked previously because the attentional selection mechanism had not yet been modeled in studies on the time course of salience.

## Introduction

“You never get a second chance to make a first impression.” This colloquial phrase aptly describes what happens to local contrasts—like color, orientation, or luminance contrasts—in the visual system. Physical contrasts like these that stand out from their surroundings are referred to as salience (e.g., Treue, 2003). Salience affects attention and hence how limited cognitive resources are distributed. The strength of this influence is contingent on the timing: Once the window of opportunity has closed, even strong contrasts cease to affect attention.

### Characteristics of salience

Evidence for a fast time course of attention has been provided several decades ago by studies using a broad variety of designs. Early evidence for such a time course of attention stems from studies using peripheral cues that were present in some conditions and absent in others. In these studies, the peripheral cue’s effect on attention was strong after 50 ms to 150 ms and declined afterward (Shepherd & Müller, 1989; Nakayama & Mackeben, 1989; Müller & Rabbitt, 1989). This corresponds with the more general idea that timing is crucial for attention. Temporal dynamics of attention can severely affect the processing of visual stimuli (for reviews see; Kinchla, 1992; Egeth & Yantis, 1997; Olivers, 2007). Thus, understanding visual attention involves understanding its temporal dynamics including its quick and transient component driven by stimuli.

The time course of salience in the first second after onset was studied by using a variety of experimental paradigms and different operationalizations (Donk & Soesman, (2010; Dombrowe, Olivers & Donk, 2010; Couffe, Mizzi & Michael, 2016; Donk & Soesman, 2011; Donk & van Zoest, 2008; van Zoest, Donk & Van der Stigchel, 2012) Silvis & Donk, 2014; van Zoest & Kerzel, 2015). Dombrowe et al. (2010) cued speeded responses with salience displays. They reported that the response time advantage for the salient stimulus is low after a presentation duration of 30 ms–60 ms, rises to a peak at 240 ms and 480 ms, and is gone after 960 ms. Another cueing study by Donk & Soesman (2010) found a response time advantage for the salient stimulus after just 42 ms. This advantage reached its peak after 158 ms and was weakened after 483 ms. Donk and Soesman (2011) used temporal order judgments (TOJs). They tested two presentation durations of the salience display affecting the subsequent TOJ: After 58 ms, the effect of salience on attention was stronger than after 800 ms. However, the effects of salience did not vanish. Instead, a strongly salient and weakly salient stimulus induced the same attentional advantage after 800 ms. Apparently, the uniqueness of the element in the display maintained an attentional advantage, but the attentional advantage from the strength of contrast disappeared over the first second after presentation. Using a visual search task, Couffe et al. (2016) found an increase in attentional advantage over the first 100 ms. The saccadic latency study by Donk and van Zoest (2008) reports an advantage for salient stimuli for short latencies in the 175 ms and 200 ms bins that declines afterward. Van Zoest et al. (2012) report that the curvature of saccadic trajectories is strongly affected by the salience of a distractor after 180 ms but that this effect vanishes after 300 ms. To sum up the different findings, the general expectation is that the attentional advantage caused by salience rises quickly. Afterward, the influence of salience declines. A unique contrast can retain a weakened attentional advantage even after 800 ms.

In general, salience directs attention based on local physical contrast. This way of orienting of attention has been widely recognized as a central component of visual attention although its independence of other influences is still a matter of debate (e.g., Wolfe, Cave & Franzel, 1989; Müller & Krummenacher, 2006; Wolfe & Horowitz, 2017; Theeuwes, 2019). There are many types of physical contrast that attract attention (Wolfe & Horowitz, 2004). The effect of visual salience on attention is not merely present or absent. The higher a contrast between stimulus and its surroundings, the stronger its attentional advantage (Duncan & Humphreys, 1989).

How much salience is caused by a particular local contrast has been studied theoretically and empirically. Computational models (e.g., Itti & Koch, 2001; Li, 2002) provide an explanation of how a salience value may arise from a cognitive process. However, these models do not render empirical measures of salience irrelevant because their predictive power varies with different operationalizations (Koehler, Guo, Zhang, & Eckstein, 2014) and they are sometimes even conflicting with empirical findings (Einhäuser & König, 2003; Onat, Açık, Schumann & König, 2014).

The need for model evaluation motivates the development of empirical salience measures. Different empirical measures of salience have been proposed (Huang & Pashler, 2005; Nothdurft, 2000; Koene & Zhaoping, 2007). In the case of several local contrasts at the same location, it is not obvious how these contrasts interact to produce overall salience. Whereas these empirical studies largely agree on qualitative aspects of salience (e.g. that the more types of contrast, the stronger the salience), they differ in their quantitative estimation of the strength of salience (e.g., on the strength of interactions of contrasts; Koene & Zhaoping, 2007; Nothdurft, 2000). A possible cause for diverging results is that each operationalization for testing quantitative hypotheses about the strength of salience is justified in a verbal argument. A formal model linking the salience measure and the operationalization may provide a better explanation as to why a physical contrast is associated with a numerical salience value (Krüger, Tünnermann, Rohlfing, & Scharlau, 2018).

Why should researchers interested in attention care about—possibly minute—quantitative differences in salience caused by physical contrast and when it is presented? The reason why even small salience differences matter is that attention is a selective process (Carrasco, 2011). In this selective process, attended stimuli have an advantage in competing for limited resources (Desimone & Duncan, 1995; Beck & Kastner, 2009; Reynolds & Chelazzi 2004). Therefore, even small differences can determine whether a stimulus is processed further to be represented consciously or whether it passes unbeknownst to the observer (Luck & Vogel, 1997; Walker, Stafford & Davis, 2008).

### Modeling salience-based selection

Understanding visual attention as a selective process that is—among other factors—driven by local contrasts and their timing results in a complex process. Formal models are particularly apt for dealing with complex cognitive phenomena (Marewski & Olsson, 2009; Rodgers, 2010). They provide good quantitative explanations (Krüger et al., 2018), have been successful in accumulating progress in attentional research in the past (Logan, 2004), and, importantly, force high specificity and quantitative precision (Luce, 1999; Taagepera, 2008).

The merits of modeling in psychology have been described by different authors (e.g., Taagepera, 2008; Rodgers, 2010; Marewski & Olsson, 2009). Models are particularly valuable in combination with Bayesian inference for understanding nonlinear cognition processes (e.g., Rouder & Lu, 2005; Lee, 2011; Van de Schoot, Winter, Ryan, Zondervan-Zwijnenburg & Depaoli, 2017). Also, models enable parameter estimation, which has arguably been undervalued in classical hypothesis tests (Cumming, 2014). Because models are more explicit in what is expected to happen than a prediction of a directed effect, they provide a more severe test of hypotheses (Rouder, Morey, Verhagen, Province & Wagenmakers, 2016) which is well in line with the hypothetico-deductive method applied in psychology (Gelman & Shalizi, 2013). Providing such a more severe test means that it is easier to potentially falsify a claim.

For attention research, cumulative progress in formal models of attention has been reviewed by Logan (2004). One of the frameworks reviewed by Logan is Bundesen’s theory of visual attention (TVA; Bundesen, 1990). TVA formally models visual selection and recognition as a parallel biased competition. The mechanism can be imagined as a race: Each stimulus in the visual field is associated with a processing speed. Only the stimuli finishing the race first are represented in visual short-term memory until its capacity is exhausted. Stimuli arriving thereafter cannot be represented for later recall. The sum of the stimuli’s processing speed is the overall visual processing capacity that represents the available processing resources for the current task. Each stimulus’ individual processing speed is affected by the relative attentional advantage of the stimulus, its attentional weights. These weights, in turn, are affected by the task relevance and the sensory evidence of the stimulus’ features (for a more detailed explanation, see Bundesen, Vangkilde & Petersen, 2015). The overall visual processing capacity and attentional weights are most important for the present paper. As will be detailed below, the TVA functions and parameters describe observed data, but also have very precise theoretical meanings.

Although salience was originally not explicitly modeled in TVA, a link between TVA’s attentional weights and visual salience had been suspected when TVA was interpreted in terms of neuronal processes (Bundesen, Habekost & Kyllingsbæk, 2005, Bundesen, Habekost & Kyllingsbæk, 2011). More recently, a salience measure, formally denoted as $\kappa$, has been added to TVA to include the influence of salience on the attentional weights and hence the quantitative contribution of salience to selection (Nordfang, Dyrholm, & Bundesen, 2013). This value functions as a common measure for salience. Such a single measure of salience has been sought before in experimental studies (e.g., Nothdurft, 2000, 1993; Huang & Pashler, 2005). Although pursuing the same goal, these studies used very different stimulus material (especially large sets of homogeneous background items) that cannot be interpreted in terms of TVA so that there is yet no direct connection between these two research strands. In a previous work, we made this connection between the stimulus material and a TVA-based formal salience measure (Krüger, Tünnermann, & Scharlau, 2016, 2017; Tünnermann, Krüger & Scharlau, 2017).

We combined TVA’s cognitive model of visual attention with a simple task that allows for uncomplicated salience-related stimulus manipulation. During the task, the temporal order of two visual events has to be judged in a so-called temporal-order judgment (TOJ). Both events are separated by a brief interval, the stimulus onset asynchrony (SOA). This accuracy-based task requires a binary decision (“A before B” or “B before A”). The task is related to attention because of the phenomenon that an attended stimulus is perceived earlier than an otherwise similar but unattended stimulus. Attention thus leads to a systematic deviation of the reported from the objectively correct order (for a review, see Spence & Parise, 2010). The TOJ allows us to investigate the time course of visual salience by manipulating the interval between the onset of a salience display and the subsequent two visual events that have to be judged. The resulting judgment can be modeled as the outcome of the general attentional cognitive processes assumed by TVA (Tünnermann, Petersen & Scharlau, 2015). Thus, while in line with previous empirical and modeling works, the TVA-based model additionally provides a formal link to a general theory of attention and its notion of salience (Nordfang et al., 2013) so that TOJ data can be explained in terms of the overall visual processing capacity and a theoretically meaningful salience measure (Krüger et al., 2016, 2017).

Originally, we merely aimed to show that the previous modeling and empirical work (Krüger et al., 2016, 2017) can be extended to measure the time course of salience in TVA’s salience measure $\kappa$. We planned Experiment 1 with five time intervals between salience onset and salience measurement with full randomization. Contrary to our expectations, only the overall visual processing capacity—the other free model parameter—was severely reduced before 150 ms. Because the result was an unexpected discovery, we conducted a replication. The only change from the original experiment was the use of a blocked design instead of a fully randomized design because this blocked design had been used in a previous TOJ time-course of salience article (Donk& Soesman 2011) and thus helps to make results comparable. Experiment 2 confirmed the discovery from Experiment 1, and both experiments suggest that the time course observed in the TOJ is affected by a difficulty to solve the task and by a change in the effect of salience on attention.

## General method

Two TVA parameters are crucial for the following experiments. These parameters are the overall processing speed or capacity, *C*, and the stimulus-driven component of attention, $\kappa$, which measures salience. This section explains why and how data from TOJs can be mathematically linked to these two parameters. The section can be skipped without loss of continuity, if the reader is either familiar with the modeling or prefers to take both parameters at face value.

In a TOJ, two stimuli are task-relevant. To distinguish them, we call them probe, *p*, and reference, *r*. These names originate from the fact that the probe, the experimental stimulus, is salient while the reference, always non-salient, serves as the control stimulus. The TOJ can be understood as the outcome of a race between these two stimuli. Whichever finishes its race first is perceived to be the first stimulus. For winning the race, processing speed matters. According to TVA, each stimulus has an associated processing rate, $v_p$ and $v_r$, that determines the speed of processing. These rates are given in stimuli per second (Hz). Their sum yields the overall processing speed, *C*, also given as a rate.

According to the extended weight equation shown in Eq. 1 (Nordfang et al., 2013), salience and goal-directed influences interact multiplicatively to produce attentional weights. The variable $w_x$ determines the attentional weight for a specific stimulus *x*. The factor $\kappa _x$ describes the effect of salience of object *x*, *R* is the set of all relevant semantic categories, $\pi _j$ determines the pertinence of the category *j*, and $\eta (x,j)$ the sensory evidence that the stimulus in question *x* belongs to the semantic category *j*. Details about these attentional parameters are described by Bundesen (1990, 1998). In the current experimental context, the goal-directed influences on the attentional weight, $\pi$ and $\eta$, are kept constant by designing a task where both stimuli are equally task-relevant and provide the same sensory evidence for the event to be judged. If both stimuli are equally relevant, only $\kappa$ makes a difference for their attentional weight. Nordfang et al. (2013) defined $\kappa _r$ to be 1 for a stimulus without any specific bottom-up salience. The two stimuli’s attentional weights can be normalized by dividing by the sum of weights to distribute the overall processing speed, *C*, to either one as shown in Eqs. 2 and 3. So, it is possible to express the processing speeds of two stimuli as a function of the overall processing speed, *C*, and the salience value of the salience stimulus, $\kappa _p$.

1 $$\begin{aligned} w_x = \kappa _x \sum _{j \in R} \eta (x,j)\pi _j \end{aligned}$$

The two processing speeds, $v_p$ and $v_r$, are connected to the data stochastically in such a way that the variables $v_p$ and $v_r$ are used as parameters for a psychometric function. Together with the stimulus onset asynchrony (SOA), i.e. the temporal delay between the two temporal events of which the order has to be judged, the TOJ can be described formally by a psychometric function. This is explained in detail by Tünnermann et al. (2015) and Krüger et al. (2016). For the present article, it is important that this psychometric function describes the observed TOJ data.

2 $$\begin{aligned} v_p&= \frac{\kappa _p}{1+\kappa _p} \cdot C \end{aligned}$$

3 $$\begin{aligned} v_r&= \frac{1}{1+\kappa _p} \cdot C \end{aligned}$$

In terms of the parameters, $v_p$, $v_r$ and SOA denoted as $\Delta t$, the probability of encoding the probe stimulus *p* first, $P_{\mathrm {p}}$, can be expressed as two equations. The Eqs. 4 and 5 define a sigmoid-looking psychometric function (for a more detailed derivation of this function from the basic TVA, see Tünnermann et al., 2015). These functions together describe the order judgment over relevant SOA delays in terms of two processing speeds. As introduced in Eqs. 2 and 3, the processing speeds can be expressed as overall processing speed, *C*, and salience, $\kappa _p$. These two parameters will be estimated in the empirical part of the present article.

4 $$\begin{aligned} P_p(v_p, v_r, \Delta t) = 1-e^{v_p |\Delta t|}+e^{v_p |\Delta t|}\left( \frac{v_p}{v_p+v_r}\right) \quad {\text {for }} \,\, \Delta t < 0 \end{aligned}$$

for negative SOAs and

5 $$\begin{aligned} P_p(v_p, v_r, \Delta t) = e^{v_r |\Delta t|}\left( \frac{v_p}{v_p+v_r}\right) \quad {\text {for }} \,\,\Delta t \ge 0 \end{aligned}$$

**Fig. 1:**
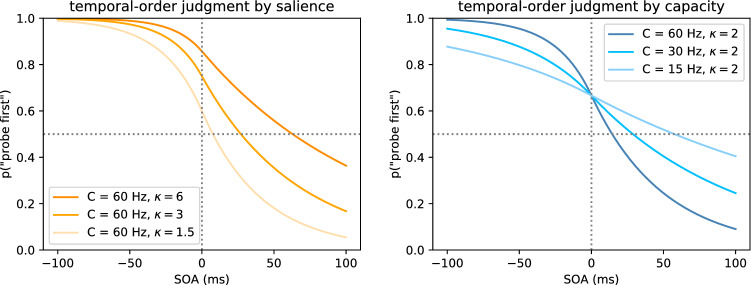
Visualization of the TOJ curve as dependent on the salience parameter, $\kappa$, and the overall processing capacity, *C*. Negative SOAs correspond to probe being shown before reference. Because a successful attentional manipulation is assumed for the present study, only functions with $\kappa > 1$ are shown ($\kappa = 1$ would mean no attentional bias and result in a function symmetrical to point (0, 0.5))

From the formalism of TVA, it is difficult to imagine how the data patterns in the TOJ depend on the salience parameter, $\kappa$, and the overall processing capacity, *C*. In Fig. 1 we provide a visualization of different $\kappa$ and *C* values within previously observed ranges. The visualized function is the psychometric function defined by Eqs. 4 and 5. The parameters *C* and $\kappa$ are converted to processing rates $v_p$ and $v_r$ by the Eqs. 2 and 3, respectively. Specifically, Eq. 4 describing how $P_p$, the probability of reporting probe first, depends on the processing rates of both stimuli and SOAs smaller than 0, whereas Eq. 5 shows this for the positive SOAs. So, roughly speaking, Eq. 4 defines the left half of the psychometric function and Eq. 5 the right half. These illustrations show that a change in salience and hence attention brings about a distinctly different change in the TOJ data when compared to the second parameter, overall processing capacity. If the overall processing capacity is low, more mistakes are made in the temporal discrimination leading to a shallow slope of the function. If salience and thus attention is changed, the same amount of processing resources is distributed differently. This distribution leads to a characteristic increase in correct discrimination of the attended stimulus, if it is indeed first, but increases the number of mistakes, if the attended stimulus is second. Independently of TVA, these patterns are in line with the basic mechanisms of visual attention. TVA, however, provides a quantitative model of the relationship between overall performance in temporal discrimination and attentional advantage.

We chose a hierarchical Bayesian model for the parameter estimation. Although there are different pros and cons to consider when using Bayesian methods (Dienes, 2011), Bayesian hierarchical models provide a range of advantages for cognitive modeling in general (Lee, 2011) and are particularly suitable for parameter estimation under a given model (Little, 2006). The relevant details of the Bayesian analysis as well as the original data and posterior predictive for model checking are given in the Appendix. Also, the analysis script is published online together with additional results and graphics (Krüger, 2020).

When evaluating the results of the following experiments, the skeptical reader may ask whether the TVA-TOJ model aptly describes the observed data. Therefore, we compare the results of the introduced model to a model using a logistic psychometric function that is commonly used in the analysis of TOJ data. Like the TVA-TOJ model, the logistic function has two parameters to describe the TOJ data through two relevant properties: the point of subjective simultaneity (PSS) and the just noticeable difference (JND) (Spence & Parise, 2010). The PSS describes the intersection with the .5 level (judging A before B as often as B before A), the latter describes a threshold indicating the precision with which the events can be judged. This function may be known to the reader as logit response function from generalized linear models, but can also be implemented in Bayesian statistical analysis (Kuss, Jäkel & Wichmann, 2005). For example, in Fig. 1, the PSS parameter can be read off at the intersection of the dotted horizontal line and the graph. Note however that—in contrast to the TVA-TOJ model—these parameters are not derived from a general theory of attention but merely descriptive.

## Experiment 1

Experiment 1 tests empirically whether salience parameter $\kappa$ depends on the display duration of a salient contrast. To this end, a salience display was presented for five durations ranging from 50 to 800 ms.

TVA’s $\kappa$ parameter is estimated from a model of the TOJ data that comprises the overall visual processing capacity *C* as a second free parameter. Whereas $\kappa$ describes the relative processing advantage of the salient stimulus, *C* describes the overall available processing resources for processing both of the stimuli.

The expectation we formulated in the introduction was that the attentional advantage caused by salience should rise quickly. Afterward, the influence of salience on attention should decline, although a unique contrast may retain a weakened attentional advantage independent of its quantitative salience value.

Building on previous studies using TVA or a TVA-based analysis of TOJ, we can formulate expectations for the parameters precisely: For healthy adults, a processing capacity of around 60 Hz is normal (Finke et al., 2005). An overall processing capacity around 60 Hz is also reported in the TVA-based analysis of TOJ (Krüger et al., 2016; Tünnermann et al., 2017). For the salience parameter, the same orientation contrast as in the present study has been measured in multiple experiments to be 2 to 2.5 (Krüger et al., 2017).

The previous results taken together lead to our hypothesis for Experiment 1: The salience parameter $\kappa$ should initially rise to around 2.5 and decrease for longer presentation durations. The overall processing capacity *C* is expected to be 60 Hz and is not expected to vary across the experimental conditions.

### Method

#### Participants

Thirty persons (15 male and 15 female; $M_\mathrm{age}= 23.27$, range 19–35) participated in Experiment 1. The size of the sample was fixed in advance and was based on earlier studies (Krüger et al., 2017). The participants were students or members of Paderborn University. Each participant gave informed written consent and reported normal or corrected-to-normal visual acuity. Participants received course credit or a payment of 8 euros per hour.

#### Apparatus

Experiment 1 was conducted using two Microsoft Windows 7 PCs and two Iiyama Vision Master Pro512 22 in. ($40.4~{\mathrm {cm}} \times 30.3~{\mathrm {cm}}$) CRT monitors (resolution $1024 \times 768$ pixels, 32-bit colors, refresh rate 100 Hz). The experimental procedure was implemented with OpenSesame (Mathôt, Schreij & Theeuwes, 2012) and PsychoPy (Peirce, 2007). The monitors were luminance calibrated by an x-rite colormunki display colorimeter. The viewing distance was 50 cm. Responses were given using the left ctrl key and the right enter key (number pad). Left and right responses were given with the left and right hand, respectively. The experiment was conducted in an experimental booth that was dimly lit.

#### Stimuli

In the beginning of each trial, a fixation cross appeared for 900 ms in the center of the screen. Afterward, a bar array of $17 \times 16$ items was shown. The size of the array comprised $34.99^\circ \times 32.93^\circ$ of visual angle. Bar length was $1.07^\circ$ and bar width $0.18^\circ$. The fixation cross replaced a bar at the horizontal center with 8 rows of bars above and 7 below. The array was drawn on a gray background, RGB (96, 96, 96) and luminance $6.98 \frac{{\rm cd}}{m^2}$ whereas bars and fixation cross were drawn in white, RGB (224, 224, 224) corresponding to $65.2 \frac{cd}{m^2}$.

Probe and reference stimulus were placed at two fixed positions on the left and on the right of the fixation cross (eccentricity $8.24^\circ$ of visual angle). The reference stimulus had the same orientation as the background elements, whereas the probe stimulus was rotated by $90^\circ$ in comparison to the background elements and hence had the maximum orientation contrast of $\Delta o = 90^\circ$. Whether probe or reference occurred on the right was balanced and randomized. The orientation of the background elements was chosen randomly.

**Fig. 2:**
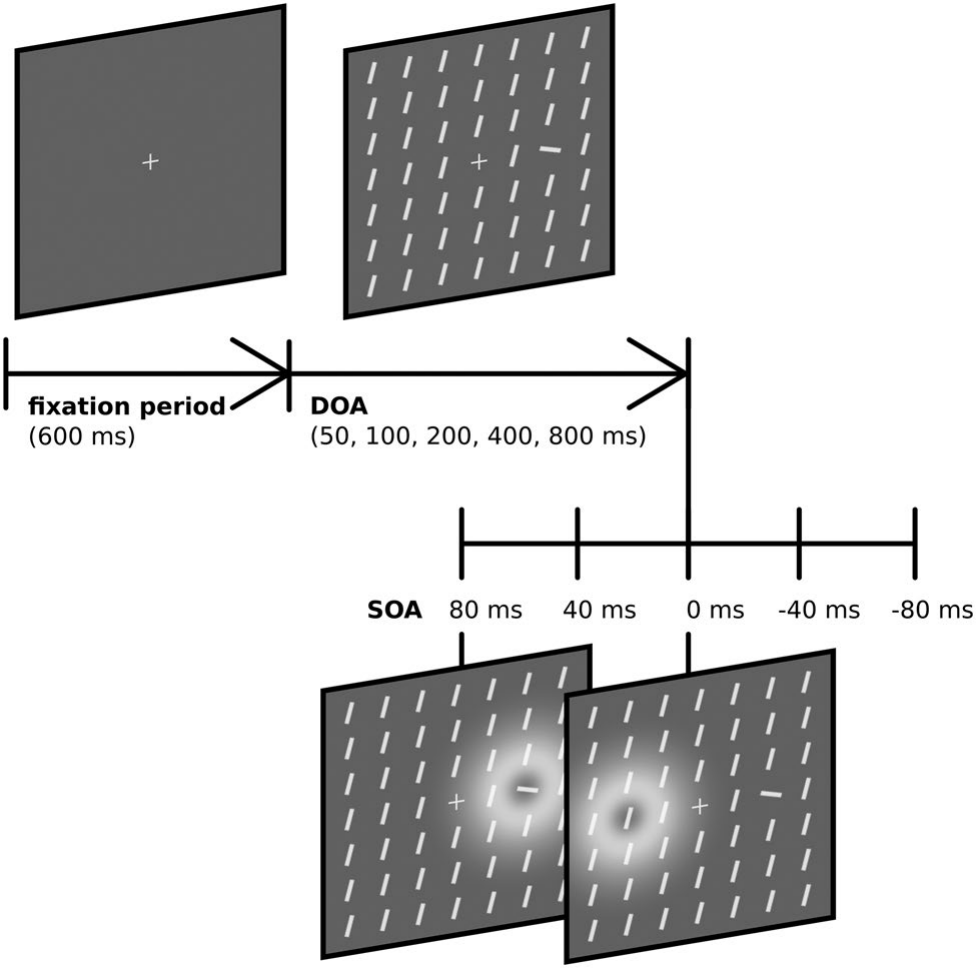
Schematic procedure of a trial. After an initial fixation period of 600 ms, the salience display is shown for a duration depending on the experimental DOA condition. Afterward, both stimuli flicker separated by a brief interval depending on the SOA. Positive SOAs correspond to probe-before-reference conditions whereas negative SOAs correspond to probe-after-reference conditions. The white corona symbolizes a rapid off- and onset that was perceived as a flicker

After a fixation period of 600 ms, the salience display was presented for a display onset asynchrony (DOA) of 50, 100, 200, 400, or 800 ms before the probe and reference stimuli flickered. The display persisted until the end of the trial—only the length of the visibility prior to the TOJ was varied. The only change to the display was the brief flicker of the probe and reference stimulus. The flicker was implemented by an offset and subsequent onset, that is, the stimulus disappeared for 80 ms. The order in which the two stimuli flickered depended on an SOA of $-80$ ms, $-40$ ms, 0 ms, 40 ms, or 80 ms, negative values meaning that the probe flickered before the reference. As an exception, in the DOA 50 condition, the 80 ms SOA could not be sampled (the reference stimulus would have to be presented before the salience display) and was therefore left out. Each SOA was presented in 34 trials except for the 0 ms SOA which was presented 68 times. The sketch in Fig. 2 shows the procedure.

#### Procedure

Instructions were presented on the screen and questions were answered by the experimenter verbally.

The participants were instructed to look at the fixation cross as soon as it became visible at the beginning of each trial. Participants then had to judge which of two flicker events was the first and respond by indicating the respective side: left or right. The response was given by pressing the left ctrl or the right enter key with the respective hand. A new trial started automatically, but breaks were offered after 50 trials. The experiment began with a training session of 40 trials. During this training, feedback about the correctness of the participant’s judgments was given. The locations of the relevant events were learned as well as the task in general. The whole session lasted approximately 45 min.

### Results and discussion

**Fig. 3:**
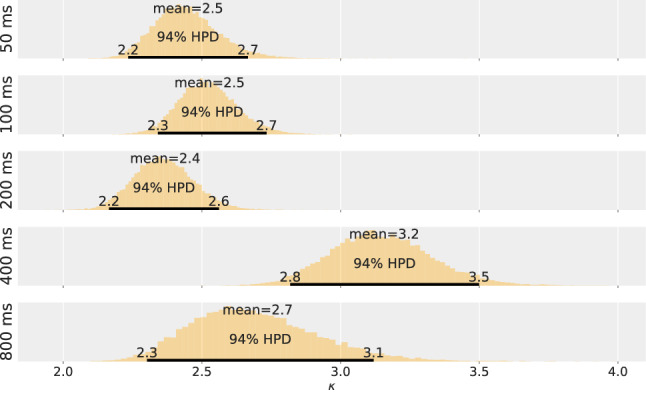
Salience estimate, $\kappa$, for the five DOA conditions of Experiment 1

**Fig. 4:**
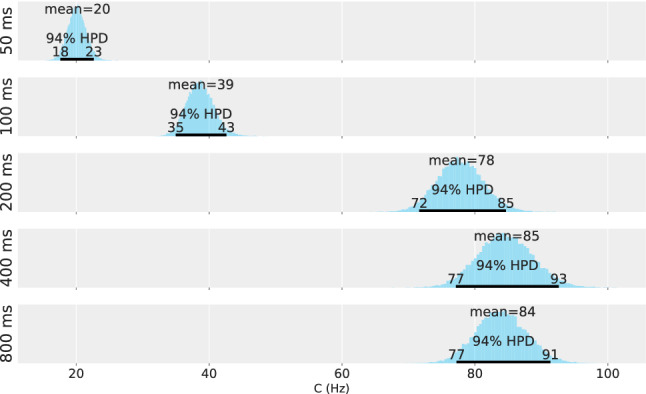
Overall processing speed estimate, *C*, for the five DOA conditions of Experiment 1

Applying the model derived from TVA, two parameters are estimated from the TOJ data for each of the five experimental conditions. A Bayesian hierarchical model is used to represent different sources of uncertainty (different repetitions, different participants, and population) according to the logic of the experimental design. The parameter estimations for the population are shown in Figs. 3 and 4—in Fig. 3 for the salience parameter $\kappa$ and in Fig. 4 for the overall processing capacity *C*.

The similarity of two groups can be assessed by comparing their parameter distributions: The smaller their overlap, the larger the effect. To provide an objective assessment of the difference of two conditions, we compute the standardized effect size (Cohen’s* d*, 1988) for each consecutive condition and compare it to a region of practical equivalence (ROPE; Kruschke, 2014) between $-0.3$ and 0.3 which is equivalent to a small effect. This constitutes a parameter-based Bayesian test (Kruschke, 2011), which checks, if at least 95% of the effect sizes that are probable after observing the data fall outside this interval so that a medium (or larger) effect is likely. If the interval of the 95% highest probability density falls completely within the ROPE, then no or a small effect is highly likely. Conversely, if this interval is completely outside the ROPE, than a medium or large effect is highly likely. This interval is reported in the square brackets. If the interval somewhat overlaps with the rope, no clear decision can be. Still, the result may be interpreted as a tendency because the maximum a posteriori estimator of the effect size (most likely effect size estimate), the number in front of the square brackets. So, roughly speaking, this procedure tests whether increasing the DOA leads to at least a medium effect between two consecutive levels. Because the effect sizes are standardized, they can be compared between different parameters, although they vary in different ranges.

The figures for salience $\kappa$ and overall processing capacity *C* depict different patterns. The salience parameter $\kappa$ is in the expected range, but does not exhibit a clear trend: The posterior distributions are largely overlapping. For the consecutive conditions, this is further substantiated by effect sizes that always overlap with the ROPE. The estimated effect sizes are $0.03 [-0.09, 0.15]$ for Condition DOA 100 ms and DOA 50 ms, $-0.065 [-0.18, 0.05]$ for Condition DOA 200 ms and DOA 100 ms, 0.28 [0.15, 0.41] for Condition DOA 400 ms and DOA 200 ms, $-0.16 [-0.33, 0.03]$ for Condition DOA 800 ms and DOA 400 ms. Thus, if there is a difference, it is rather small.

Although hypothesized to be constant, the processing capacity *C* rises steeply during the first 200 ms. The non-overlapping posterior distributions suggest that the 50 ms and 100 ms conditions are profoundly different from each other. This is again substantiated by the first two effect sizes that fall completely outside the ROPE. The estimated effect sizes are 0.86 [0.65, 1.1] for Condition DOA 100 ms and DOA 50 ms, 1 [0.83, 1.2] for Condition DOA 200 ms and DOA 100 ms, $0.14 [-0.07, 0.35]$ for Condition DOA 400 ms and DOA 200 ms, $-0.01 [-0.23, 0.2]$ for Condition DOA 800 ms and DOA 400 ms. Hence, a medium or larger effect is highly likely to occur between the first three levels of DOA. The value of the processing capacity parameter *C* reaches the expected value only after 200 ms but appears to be constant from there on.

According to the hypothesis, the $\kappa$ value should explain a difference between conditions. To check this, we formulate and test alternative models. First, we check whether there is a time course in salience parameter $\kappa$. We compare the model that yielded the parameter estimates to a model that assumes the same $\kappa$ value for all conditions i.e. that $\kappa$ does not change with the progression of time. This model is called the fixed-$\kappa$ model. Also, we test whether or not overall processing capacity stays constant (although given the parameter estimates, it seems extremely likely that this is false). The respective model is called the fixed-*C* model. We use the leave-one-out Information Criterion (looIC; Vehtari, Gelman & Gabry, 2017) that accounts for model complexity and for which small numbers indicate a better model. This comparison yields an looIC of 3896.31 for the original model, an looIC of 4019.54 for the fixed-$\kappa$ model, and an looIC of 4781.0 for the fixed-C model. For the hypothesis, this ranking means that $\kappa$ does vary over time because fixing $\kappa$ results in a worse model. Also, *C* exhibits a time course. Fixing the *C* parameter however results in a worse model than fixing *k*. Thus, we can conclude that a time course of salience exists, although the time course of *C* is more important for the observed data pattern.

Next, we compare the present model to an alternative that replaces the TVA-based psychometric function with the logistic function while retaining the same hierarchical structure. Again, we use looIC for comparison. This procedure yields an looIC of 3896.31 for the original model and an looIC of 3924.89 for the logistic function model. Priors for the logistic function model are taken from a study by Krüger et al. (2016) who provided a logistic-function-based analysis along with the TVA-based TOJ analysis. For the sake of transparency, note that the comparison of models with different parameters can depend heavily on the priors used. Therefore, we tested the robustness against different priors. It was, for example, possible to revert the order by extending the $\kappa$ prior of the TOJ-TVA model to implausible values of smaller than 1. So, a more elaborate comparison would be needed to argue for a general superiority of the fit of the TVA-TOJ model. However, we can reject the hypothesis that the analysis merely reflects an inability of the TVA-TOJ model to adequately describe the data because it is the better model for the current analysis.

Although the analysis shows that assuming no change in the salience parameter $\kappa$ is inappropriate, its change over the first second after presentation is small. Non-salient elements have a $\kappa$ value of 1 (Nordfang et al., 2013) and the estimated differences vary between 0.08 and 0.8, see Fig. 3. In comparison to the salience difference of many orientation contrasts, these differences are rather small (Krüger et al., 2017). How does such a small difference fit with previous research? The study by Donk& Soesman (2011) is closely related to the present research because they use the same experimental paradigm, the TOJ. Unfortunately, they do not report the typical descriptions of a TOJ curve which is the PSS and the JND. However, when looking at the data of Donk & Soesman (2011), the slope corresponding to JND is visually twice as shallow for the 58 ms condition in comparison to their 800 ms condition. These patterns are also visible in the present TOJ data. Donk and Soesman interpret all variance in terms of attention which is justified by their experimental design. Although mathematically not equivalent, in the present model, *C* determines how steep the TOJ curve is, whereas $\kappa$ is similar to PSS in the regard that both describe shifts of the function (although the shifts themselves are different from each other). We found a similarly striking difference between our 50 ms and 800 ms conditions as Donk and Soesman found between their 58 ms and 800 ms conditions. Although the data patterns are similar, interpretation is different: If these data are linked to the TVA model of visual selection, then it is rather overall visual processing capacity than attention that changes. Nevertheless, the aforementioned cueing studies and studies based on saccades suggest much more variability due to salience, which does not fit with the present TVA-based analysis.

Previous studies on the time course of salience used a blocked design (e.g. , Dombrowe et al., 2010; Donk & Soesman 2011). An explanation for the small variance in the salience parameter may be the fully randomized design because it may cause an equal temporal expectation for each trial, independent of its actual display duration. In the design of Experiment 1, the expectancy value for the display duration was 310 ms. Temporal expectations can severely affect processing speed (Vangkilde, Coull & Bundesen, 2012), and thus may have distorted the parameter estimation.

To sum up, we applied a model derived from a theory of visual selection, the TVA, to an experimental design, the TOJ, that is used to investigate the time course of visual salience. However, the overall ability to perform the TOJ, measured by the visual processing capacity, exhibits a stronger time course than the salience parameter. This result contradicts the hypothesis that only the distribution of attention caused by salience is affected by a time course. This finding does not originate from applying an inappropriate model to the data. This cause has been ruled out by a model comparison between the TVA-based model and a model that uses the common logistic function instead of the function derived from TVA. Thus, from the TVA perspective, there is rather a time course in the accuracy of the TOJ than in the relative advantage of the salient over the non-salient stimulus. To preclude the possibility of a chance finding or a biased parameter estimation because of temporal expectancy, we conducted a blocked replication study in Experiment 2.

## Experiment 2

Experiment 2 was conducted to replicate the findings on processing capacity *C* in Experiment 2 with blocked conditions. A blocked design makes the experiment more comparable to previous studies on the time course of salience that used blocked designs (e.g. Dombrowe et al., 2010; Donk & Soesman, 2011) and prevents temporal expectations corresponding to the mean of the DOAs that can severely influence processing speed (Vangkilde et al., 2012). The blocked design should lead to a clearer trend in the salience parameter $\kappa$. Based on this change of design and the previous parameter estimation, the hypothesis is that $\kappa$ declines over time and processing capacity rises up to 200 ms and stays constant afterward.

### Method

#### Participants

Thirty persons (9 male and 21 female; $M_\mathrm{age}= 23$, range 18–29) participated in Experiment 2. The conditions were as in Experiment 1. Participant 17 was excluded from the final analysis because they answered “probe first” independently of the SOA for last three blocks. During the first and part of the second block, the task was carried out correctly (judging temporal order). The data of Particpant 17 is available in the published data (Krüger 2020).

#### Apparatus

The same apparatus as in Experiment 1 was used.

#### Stimuli

Stimuli were the same as in Experiment 1.

#### Procedure

The procedure was the same as in Experiment 1 except that the DOA conditions appeared blocked. The order of blocks was randomized.

### Results and discussion

**Fig. 5:**
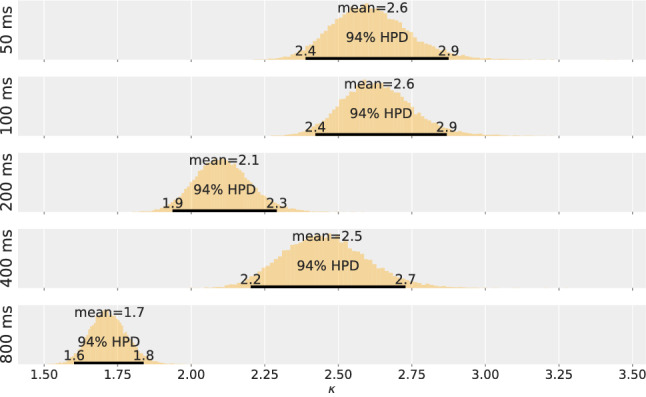
Salience estimate, $\kappa$, for the five DOA conditions of Experiment 2

**Fig. 6:**
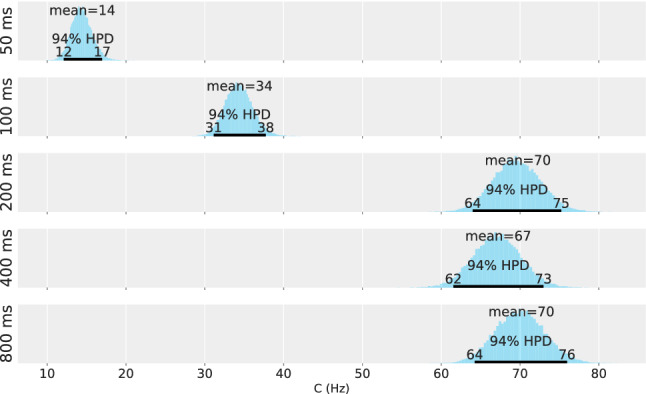
Overall processing speed estimate, *C*, for the five DOA conditions of Experiment 2

The estimated salience value, $\kappa$, is shown in Fig. 5, and the estimated overall processing capacity is shown in Fig. 6. Again, the parameter difference distributions were tested against a ROPE, see the distributions on the right. The estimations show a pattern comparable the one in Experiment 1 for both variables: Over the course of 800 ms, salience varies. However, a more pronounced time course is exhibited in the overall processing capacity. In contrast to Experiment 1, these patterns were expected and support our hypothesis by replicating the original finding.

The testing for medium or larger effects for two consecutive levels of DOA shows the same result: There is a medium or larger effect for *C* between the first three levels of DOA. The exact estimates for the capacity effect sizes are 1.1 [0.81, 1.4] for Condition DOA 100 ms and DOA 50 ms, 0.95 [0.78, 1.1] for Condition DOA 200 ms and DOA 100 ms, $-0.05 [-0.22, 0.13]$ for Condition DOA 400 ms and DOA 200 ms, $0.06 [-0.12, 0.24]$ for Condition DOA 800 ms and DOA 400 ms. The effect sizes for $\kappa$ are $0.01 [-0.12, 0.13]$ for Condition DOA 100 ms and DOA 50 ms, $-0.22 [-0.33, 0.1]$ for Condition DOA 200 ms and DOA 100 ms, 0.15 [0.02, 0.28] for Condition DOA 400 ms and DOA 200 ms, $-0.35 [-0.46, -0.23]$ for Condition DOA 800 ms and DOA 400 ms.

Comparing the looIC of the original model, 3831.49, against the fixed-$\kappa$ model, 4160.20, and fixed-*C* model, 4781.35, shows the same order as in Experiment 1: The parameter $\kappa$ is best assumed to vary between conditions. However, the parameter *C* varies more distinctly between conditions. Also, the TVA-based model was, as in Experiment 1, compared to a model using the logistic function as psychometric function while preserving the same structure. The two models were compared by looIC. This comparison yielded 3831.49 for the original and 3848.23 for the logistic function model. As in Experiment 1, this comparison stresses the point that the TVA-based model provides a good description of the data.

The parameter $\kappa$ shows more variance than in Experiment 1, which is likely due to the blocked design. A trend in the parameter value becomes more clear: The value is declining or constant with the exception of the 400 ms condition in which the estimate is higher than a strict decline would allow. This is, however, neither unprecedented nor implausible. Previous studies found an initial rise (Couffe et al., 2016) and a later peak in the time course of salience (e.g.; Dombrowe et al., 2010; van Zoest et al., 2012). This is not implausible because salience is initially processed in a feed-forward way (Li, 2002). However, receptive fields linked to salience can increase their firing rate by a delayed response enhancement based on a recurrent network. This response enhancement has been reported to take more than 200 ms (Lamme & Roelfsema, 2000; Fecteau & Munoz, 2006).

Nevertheless, the clearest evidence for a time course was again found in the overall processing capacity *C*. After the successful replication of Experiment 1, it is appropriate to interpret this distinct pattern, which corresponds to reduced resources for the task in general, when the DOA is smaller than 200 ms. One explanation of a changing processing capacity may be alertness. TVA research shows that an alerting stimulus preceding the task-relevant stimuli may indeed affect processing capacity. However, processing capacity has been reported to increase under these circumstances (Matthias et al., 2010). Besides alertness, temporal expectancy, too, is known to affect overall processing capacity (Vangkilde et al., 2012). However, the blocked or fully randomized design does not change the observed pattern.

Beyond the TVA literature, Yeshurun & Levy (2003) report that spatial attention can reduce temporal resolution. This effect has also been found to affect the temporal discrimination performance in TOJ adversely (Hein, Rolke & Ulrich, 2006). The adverse effects of a salience display could, however, not have been foreseen because Yeshurun & Levy (2003) showed that a peripheral cue limited to a specific location reduced the temporal resolution at this location. If the cue expanded in size to span the whole screen, temporal resolution would be significantly better. Also, Hein et al. (2006) used a peripheral cue limited to the location of the TOJ. Both studies concluded that spatial attention in general diminishes temporal resolution. Thus, this may explain the decrease in processing capacity *C* because a decrease in processing capacity *C* corresponds to a decreased performance in the temporal judgment.

However, Experiment 1 and Experiment 2 were not designed to test how TVA parameters relate to specific or even conflicting neurophysiological or behavioral findings beyond the recurrent patterns in the time course of salience. Thus, the main contribution of Experiment 2 is that the original findings have been successfully replicated. That is, a model derived from TVA to provide a quantitative measure of visual salience can also capture the time course of salience in theoretically interpretable variables. This model revealed a time course in the general performance of judging temporal order as well as in the relative advantage of the salient stimulus.

## General discussion

Previous studies show that the effect of visual salience on attention depends on a time course as well as physical contrast. We formally quantified the strength of salience by modeling the relationship between salience, attention and visual selection based on a theory of visual selection: Bundesen’s TVA (for the measurement of physical contrasts see Krüger et al., 2017). The TVA modeling implies two free parameters: visual salience, $\kappa$, and overall visual processing capacity, *C*. The resulting two-parameter model links data and theory mathematically: The numerical values of salience and overall processing capacity are, on the one hand, statistically inferred from the observed data. On the other hand, these two parameters have a specific theoretical meaning in a formal theory of visual attention. The present modeling and empirical approach yields a common salience measure that quantifies the effect of time course on visual salience.

In the empirical part of this article, the two-parameter model-based analysis revealed that salience is highest after 50 ms and changes over time, which was checked by a model comparison to a model assuming a fixed $\kappa$ value for all conditions. Although salience declines, the salient stimulus still receives nearly twice as much attention after 800 ms as the non-salient reference stimulus. Additionally, our findings revealed a distinct pattern in the overall processing capacity that corresponds to the general performance in the TOJ: Overall processing capacity is lowest after 50 ms and rises up until 200 ms. After that, it stays constant and is in line with the usually reported processing capacity of healthy participants (Finke et al. 2005). This finding is supported by testing for medium or larger effect sizes: Only the processing capacity *C* shows medium or large effects for consecutive DOA levels in the range up to 200 ms. This pattern means that temporal discrimination performance is strongly reduced for short display durations. Because such a pattern was not part of the hypothesis in Experiment 1, we conducted a replication in Experiment 2 which confirmed the finding.

Before the results of these parameter estimations are compared to previous research on the time course of visual salience, it is useful to take one step back and discuss the model. Formal modeling has often been advocated as important particularly for linking theory and data (e.g., Rouder, Morey & Wagenmakers, 2016; Marewski & Olsson, 2009; Taagepera, 2008; Krüger et al., 2018). Following this approach, formal models help to accumulate progress on visual attention (Logan, 2004) and provide testable predictions (Taagepera, 2008; Luce, 1999). On the other hand, as the statistician Box (1976) put it, “all models are wrong” in the sense that each model is a particular simplification of a set of phenomena guided by the principle of parsimony. Therefore, it is more appropriate to ask whether a model is useful than to ask whether it is correct. A model is only useful on a particular level of abstraction (Marr, 1982). Thus, the crucial question regarding the present model is whether it describes the phenomena adequately for its level of abstraction and whether it is useful for theorizing and accumulating results beyond individual experiments.

TVA models visual selection and attention based on the simple yet evocative concept of biased competition (Desimone & Duncan, 1995). More precisely, TVA assumes a fixed-capacity independent-race model (e.g., Shibuya & Bundesen, 1988). For the TOJ, assuming a fixed-capacity independent-race model formally implies a specific psychometric function with two parameters (Tünnermann et al., 2015). This function is defined formally by the Eqs. 4 and 5. For both experiments, we showed that using the common logistic function (Wichmann & Hill, 2001; Kuss et al., 2005) to describe TOJs results in a worse model when compared to the TVA-based psychometric function. A caveat, however, is that we cannot provide a systematic comparison that would involve all commonly used psychometric functions, data from more than two experiments, and more elaborate choice of priors. Thus, we do not claim a general superiority of the TVA-based psychometric function but merely that the model provides a fit comparable to commonly used models. This assessment is in line with the visually similar posterior predictives of the TVA-based and the logistic functions (Krüger et al., 2016; Krüger, 2020). Overall, a specific theoretic meaning of parameters is gained while an adequate description of the data is maintained.

The specific meaning of TVA’s salience parameter allows theorizing about stimulus-driven attention beyond the TOJ design. The salience parameter was developed from a partial-report design (Nordfang et al., 2013) and research on it continues within this experimental design (Nordfang, Staugaard, & Bundesen, 2017). This line of research spells out the previously supposed connection between TVA and salience research (Bundesen et al., 2005, 2015). Whereas salience models (e.g. Koch & Ullman, 1985; Li, 2002) and particularly their implementation as computational models (for a survey see Frintrop, Rome & Christensen, 2010) provide a quantitative prediction of a particular stimulus’ salience, TVA has a clear prediction of how this value should affect visual selection. This prediction is used to infer salience from the process of visual selection occurring in TOJ (Krüger et al. 2017) as well as report-based designs (Nordfang et al., 2013, 2017).

To sum up our model evaluation, the model provides a good fit to the TOJ data and contributes to the integration of salience and TVA research. We hope that the reader is inclined to entertain the presented model as a useful way of looking at attentional influences of salience in TOJ as modeled by TVA so that the parameter values can be understood as representative of the properties of cognitive processes.

The salience parameter estimation provides an estimation of the time course of salience. Whereas the literature agrees that a time course of salience exists, there is discord about its shape. The different shapes of the time course have been reviewed in the introduction to form the original hypothesis. The reviewed time courses can be summarized in two points: First, does the effect of salience on attention increase during the presentation duration up until 200 ms (e.g., Dombrowe et al., 2010; van Zoest et al., 2012) or is its maximum reached after a short duration of approximately 50 ms (the latter finding is not as often reported as the first one; Donk& Soesman 2011; Donk & van Zoest, 2008)? Second, do effects from salience vanish after a few hundred milliseconds (e.g., Dombrowe et al., 2010; van Zoest et al., 2012) or is the attentional advantage of a salient stimulus longer-lasting (Donk & Soesman, 2011)? Interestingly, the TOJ study by Donk and Soesman, whose data strongly resembles our TOJ data, deviates from the speeded response studies. The deviation may be explained by the fact that the TOJs are accuracy-based whereas other studies involve speeded motor responses (e.g., no early advantage of salient stimuli was shown by van Zoest & Kerzel, 2015). If a quantitative estimate of the salience effect on attention and thus visual selection is desired, a measure not affected by motor components seems apt.

It is important to note that our evaluation of the strength of salience works exclusively through the surrogate of attention. An operationalization using perception (e.g., Nothdurft, 2000) may arrive at different results and we do not make any statements about a potential change of the perceptual properties of the salient stimulus (e.g., Kerzel, Schönhammer, Burra, Born & Souto, 2011).

It is not optimal that one SOA is missing in the 50 ms DOA condition. However, in comparison to our earlier works, we already reduced the SOA from a maximum of 100 ms to 80 ms to be able to use all SOAs in the 100 ms DOA condition. Balanced SOAs for the 50 ms DOA condition were not possible. Yet, we wanted to include this condition because DOAs in this region have been used in the literature before. Note that the Bayesian analysis accounts for the reduced amount of data in the 100 ms DOA condition. This results in wider posterior distributions, if everything else is kept constant. Therefore, the narrow parameter distribution for this condition must originate from less variance in the available data. Yet, we cannot exclude the possibility of a bias in this condition caused by the unbalanced design. However, if the 50 ms DOA condition were removed altogether, the conclusion would still hold because of the 100 ms DOA condition’s difference to the longer DOA conditions.

Another limiting factor of the study is that some of TVA’s attentional parameters cannot be estimated: As of yet, it is not possible to derive the minimum effective exposure duration, $t_0$, with a TOJ-based design because the two parameters are assumed to cancel each other out (for a detailed explanation, see Tünnermann et al., 2015). This prohibits a comparison within condition (probe against reference) but also a comparison of possible changes in $t_0$ across conditions. While it may be unsettling that there are potential causes that cannot be detected by the present model, this may be addressed by developing a model specifically designed to discern such influences in future research.

**Fig. 7:**
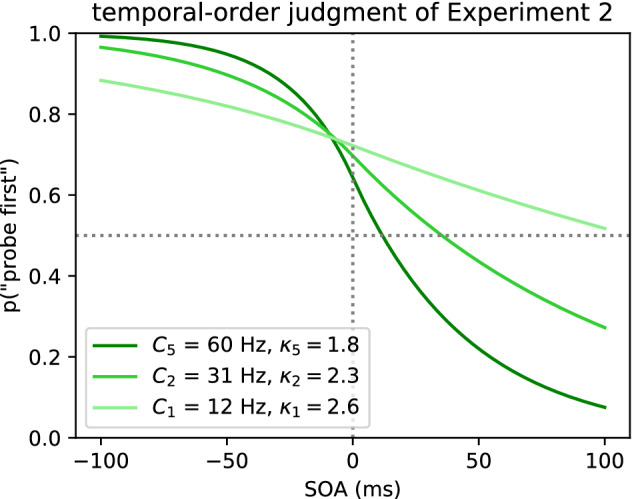
Visualization of the TOJ curve as dependent on the salience parameter, $\kappa$, and overall processing capacity, *C*, in Condition 1 (50 ms), 2 (100 ms), and 5 (800 ms) of Experiment 2

Despite its limitations, our theory-based approach made a specific prediction of how salience should affect the data, which we illustrated for the salience parameter and for the overall processing capacity in Fig. 1. Based on the literature, we assumed that only salience, i.e. the attentional advantage, would change for different DOAs. This prediction has been disproved by Experiment 1: In addition to the change in the salience parameter, we found a distinct increase in the overall processing capacity. An overall processing capacity change corresponds to a change in temporal discrimination performance. Poor temporal discrimination performance results in a flat TOJ curve. This pattern is found in the curves belonging to the actual parameter estimates (maximum a posteriori probabilities) of Experiment 2. Figure 7 shows the curves belonging to the estimates for Condition 1 (50 ms), 2 (100 ms), and 5 (800 ms) of Experiment 2. Such a flat pattern for short delays between display onset and SOA was also present in the data of Donk and Soesman (2011). Without considering this change in the overall performance, i.e. slope of the curve, the salience estimate may be confounded by the performance in the TOJ.

Within the scope of this article, we cannot investigate whether or not only temporal discrimination performance is reduced until 200 ms after the onset of the salience display. Peripheral cues have been reported to diminish temporal discrimination performance (Yeshurun & Levy, 2003; Hein et al., 2006). Attempts have been made to explain temporal dynamics with TVA theoretically (Schneider 2013) for sequences of saccades and by means of experimentation and modeling of attentional dwell time (Petersen, Kyllingsbæk & Bundesen, 2012). Attentional dwell time may seem like a possible explanation of a reduced processing capacity: After the appearance of a visual stimulus, resources are bound to this stimulus and are not available for the processing of other stimuli that appear afterward. However, it has been shown that task-irrelevant stimuli (much like the task-irrelevant salience display) do not bind resources similarly. Only if a target precedes another target, resources are reduced (Olivers, 2007). Thus, it is not obvious whether or not the task-irrelevant onset of the salience display binds processing resources in the same way as described by this research. (Tünnermann & Scharlau, 2016) examined irrelevant peripheral cues in TOJs using a TVA-based analysis. This approach revealed that peripheral cues affect the overall processing capacity by being erroneously encoded as the target. This result hints at the possibility that the onset of the TOJ-relevant bar in the salience display can be confused with the relevant temporal event. If so, this should not only depend on the flicker (off- and onset) used in the present study: Donk and Soesman (2011) used a color change instead of the flicker, and they also reported a flatter pattern for short display durations. Whether the onset of the salience display can be confused with the task-relevant events, however, remains to be tested empirically.

Low C values for the short DOA conditions might be an artifact caused by the flicker. At least presentation duration and perceived offset are negatively proportional in the range used (Coltheart, 1980). However, even in these conditions participants’ accuracies were far from guessing probability (which would be a constant function of SOA). Nevertheless, there may have been a chance of not seeing the offset. Whether or not this also impairs the detection of the re-onset 80 ms later is not certain. Also, Bloch’s law might indicate the flicker as a potential confound. However, Donk and Soesman (2011) already reported a reduced slope for the short DOA condition in both experiments (see Figs. 2 and 3 from their publication). Because there was no offset involved, it cannot explain this pattern that would be visible in a reduced difference limen when fitting psychometric functions (Kuss et al., 2005; Wichmann & Hill, 2001) or a reduced *C* in the TVA based model, see Fig. 1. Thus, the data pattern is not new, but merely unexplained as of yet.

A model-based salience measure does not come without its caveats: Because TVA-designs are accuracy-based, it is difficult to relate the estimated salience value to salience research using speeded responses. One way of reconciling these lines of research is by referring to TVA models that also model response times (Kyllingsbæk, Markussen & Bundesen, 2012; Blurton, Nielsen, Kyllingsbæk & Bundesen, 2016). These models are, however, more complex, and it remains to be seen, if they can be used to estimate the salience parameter $\kappa$. Also, the salience value is only meaningful, if the modeling of TOJ as an capacity-limited independent-race model and more broadly biased competition (Desimone & Duncan, 1995) is accepted. If this view is rejected, the salience value must be rejected as well.

To sum up, we measured the time course of salience successfully with a model derived from a theory of visual selection, TVA. We further showed that the model adequately describes the observed data by a comparison to a common psychometric model. This analysis revealed that salience decreases over time but remains an influence on attention even after 800 ms. However, the performance in the TOJ was much worse for short display durations of 50 ms and 100 ms. This effect is captured by overall processing capacity. Thus, the salience measure requires accepting basic TVA modeling but enables researchers to sharpen the concept of visual salience by differentiating actual influences on salience from possible confounds.

## Figures and Tables

**Fig. 8:**
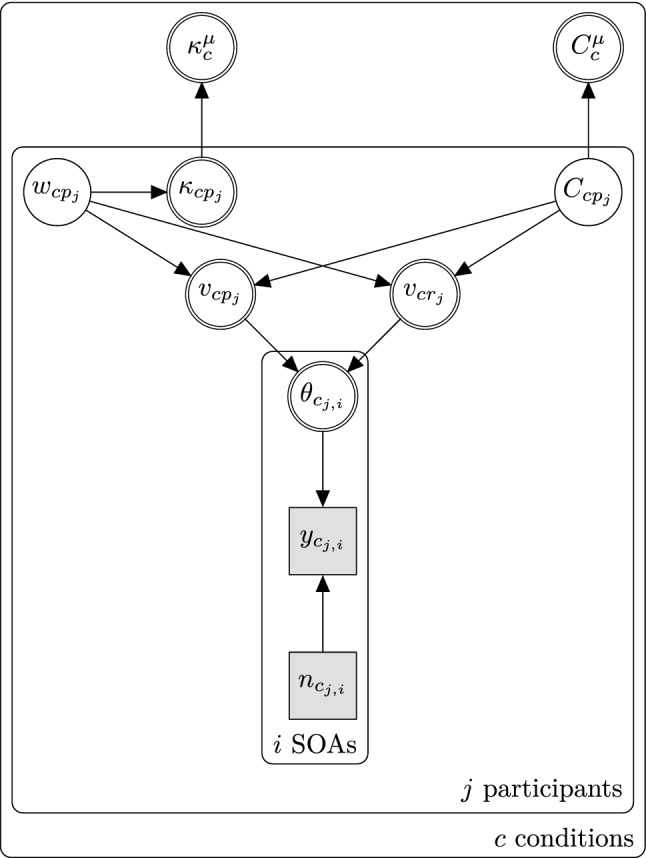
Hierarchical Bayesian graphical model connecting TOJ data given by *y* and *n* with TVA parameters *C* and $\kappa$ (salience). Each condition is treated as independent. The index *i* indicates the SOAs within a condition, *j* indicates the participant, and *c* indicates the condition of the experiment

**Fig. 9:**
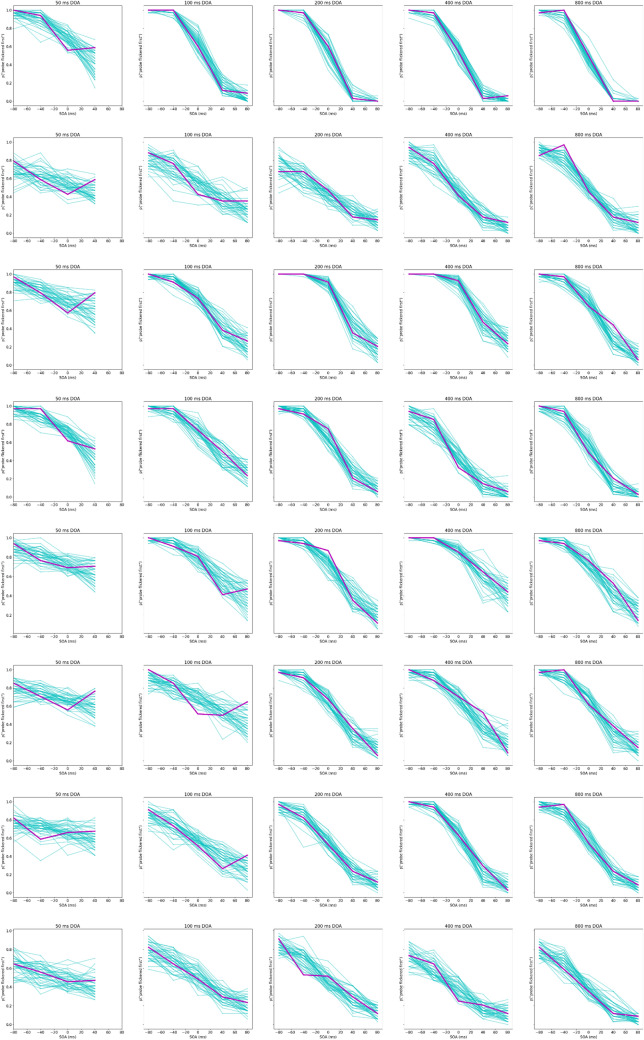
Posterior predictive of success chance (cyan) and observed success chance (purple) for Experiment 1 and the TVA-TOJ-model Participants 1–8 are shown exemplarily (for all plots, see the published data) (color figure online)

**Fig. 10:**
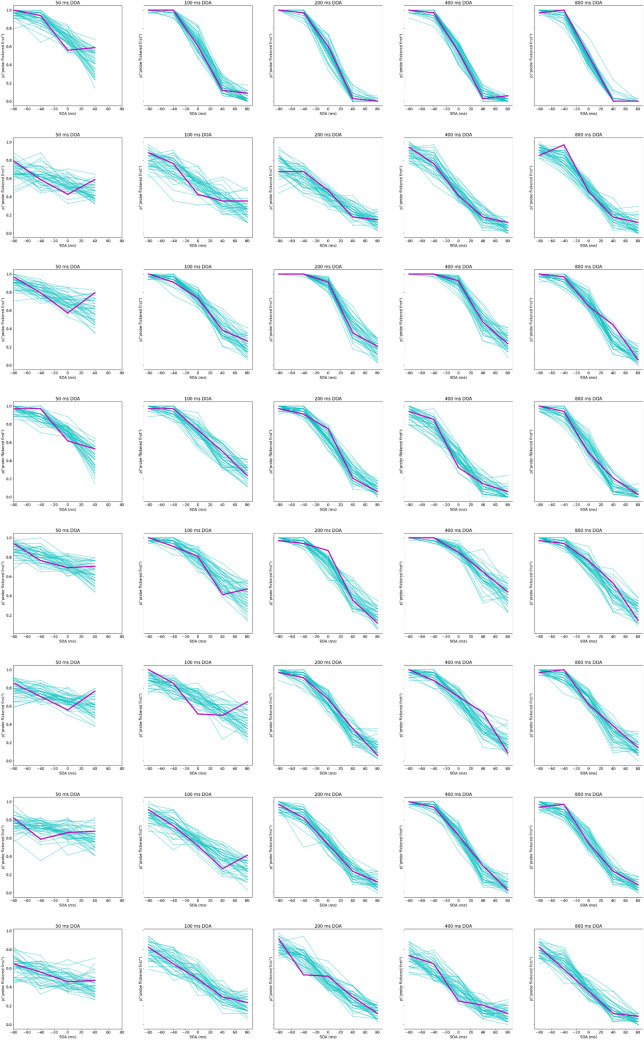
Posterior predictive of success chance (cyan) and observed success chance (purple) for Experiment 2 and the TVA-TOJ-model Participants 1–8 are shown exemplarily (for all plots, see the published data) (color figure online)

## Appendix A

### Details of Bayesian models and analysis

The data from the TOJ were analyzed by a hierarchical Bayesian model so that individual parameters were estimated and their means were used as a group estimate. The corresponding model is depicted in Fig. 8 and Tables 1, 2. The resources for modeling and analysis were provided by Kruschke (2014) and Lee and Wagenmakers (2014). The model has been implemented with pymc3. The used script is published online (Krüger, 2020) together with detailed graphics and results for all used models.

For the data analysis, the NUTS sampler has been used with 5000 samples in each of four chains excluding 500 samples for burning. We checked the convergence of the sampling process by the diagnostics provided by pymc3.

**Table 1:** Variables of the the model

Variable	Explanation
$\kappa _{\mathrm{cp}_j} = w_{cp_j}/(1-w_{\mathrm{cp}_j})$	Salience of the probe
$C_{c}^\mu = \hbox {mean}(C_{\mathrm{cp}_j})$	Mean *C* per condition
$v_{\mathrm{cp}_j} \leftarrow C_{s_j} \cdot w_{\mathrm{cp}_j}$	Participant processing rate (probe)
$v_{\mathrm{cr}_j} \leftarrow C_{s_j} \cdot (1-w_{\mathrm{cp}_j})$	Participant processing rate (reference)
$\theta _{s_{j,i}} \leftarrow P_A(v_\mathrm{sp}, v_\mathrm{sr}, {\mathrm {SOA}})$	Probability of “Probe first”
$y_{c_{j,i}} \leftarrow binomial(\theta _{s_{j,i}}, n_{s_{j,i}})$	Count “Probe first”

**Table 2:** Prior distributions of the model

$$w_{\mathrm{cp}_j} \sim {\mathrm {dunif}}(0.5,1)$$	
$$C_{\mathrm{cp}_j} \sim {\mathrm {dunif}}(0,0.25)$$ (in kHz)	

## Appendix B

### Posterior predictive

In this section we show the posterior predictive of the model as well as a summary of the original data per participant (Figs. 9, 10).
